# Lifestyle, reproductive factors and food intake in Greenlandic pregnant women: The ACCEPT – sub-study

**DOI:** 10.3402/ijch.v74.29469

**Published:** 2015-11-17

**Authors:** Ane-Kersti Skaarup Knudsen, Manhai Long, Henning S. Pedersen, Eva Cecilie Bonefeld-Jørgensen

**Affiliations:** 1 Centre for Arctic Health & Unit of Cellular and Molecular Toxicology, Department of Public Health, Aarhus University, Aarhus, Denmark; 2 Primary Health Care Center, Queen Ingrid Hospital, Nuuk, Greenland

**Keywords:** food frequency questionnaire, diet, pregnancy, Greenland, reproductive health

## Abstract

**Background:**

In the past decades, Greenland has changed from a hunter society to a more western lifestyle, causing less intake of traditional food, such as marine mammals, fish and seabirds. These changes in the living conditions and food habits might impact the maternal health in Greenland.

**Objectives:**

To describe lifestyle, reproductive factors and food intake in Greenlandic pregnant women, and to assess possible age and geographical differences.

**Design:**

Cross-sectional study of 189 Greenlandic pregnant women. Inclusion criteria were ≥18 years and lived >50% of their life in Greenland. Data were collected in 2010–2011, and information was obtained from lifestyle and food frequency questionnaires. Two age groups for comparison were given for the pregnant women (<27 years vs. ≥27 years) with regard to the median age. Region groups for comparison were West, Disko Bay, South, North and East.

**Results:**

Population characteristics showed that 43.3% had pre-pregnancy body mass index (BMI) >25.0 kg/m^2^, 46.3% were current smokers in the beginning of their pregnancy and few participants consumed alcohol during pregnancy. Women <27 years were more in doubt regarding planned breastfeeding period and consumed more dried fish and fast food. A trend for higher alcohol intake during pregnancy was found for women ≥27 years. The regional differences showed that women living >50% in North, South and West had a higher alcohol intake during pregnancy. Women in North had the fewest breastfeeding plans. Women in Disko Bay had the lowest intake of terrestrial species. No significant geographical differences were found for intake of marine mammals or seabirds.

**Conclusions:**

The present study found relatively high BMI level and high smoking frequency in Greenlandic pregnant women. Age and region differences were found for alcohol consumption, breastfeeding plans and food intake profile. Further research is needed to implement relevant maternal health intervention programs in Greenland.

For many centuries, the indigenous Inuit people lived isolated and undisturbed by Western civilization. In the past decades, the Inuit have experienced substantial transitions from a hunter society to a more western community causing changes in lifestyle, such as social, cultural and economic factors. These changes influence the health status in Greenland, including maternal and child health (1,2). The westernization of Greenland have led to a more sedentary lifestyle and reduced the intake of traditional marine food and increased the intake of imported market food. The traditional Inuit food remains an important cultural, spiritual and social connection to the surrounding nature. In addition, the traditional food is a significant nutritional source for proteins, vitamins, *n*-3 polyunsaturated fatty acids and antioxidants (3–5).

Greenland was assumed to be a pristine and unpolluted area. However, environmental contaminants are transported over long distances through the atmosphere and ocean currents to the Arctic. The contaminants are ubiquitous, have long half-lives and biomagnifying in the environment, the marine food web and humans. The contaminants include heavy metals and persistent organic pollutants (POPs) (6–8). Some contaminants are passed from mothers to the foetus through the placenta barrier and to the child by breastfeeding (2,6). Exposure to environmental contaminants such as POPs through intake of traditional food has been related to have adverse health effects on the immune-, neuro-, reproductive, and endocrine system as well as increased risk of some cancers (6,7). The dilemma about the benefits and disadvantages of traditional food is termed “The Arctic dilemma” (2).

Pregnant women and women in the childbearing age are high-risk groups regarding health aspects for future generations. Main factors presumed to have an impact on maternal health are prenatal care, genetic, lifestyle, education level and food intake. Prenatal care is a major predictor of pregnancy outcome, and approximately 90% of the mothers in Greenland receive complete prenatal care (1). Screening, therapeutic interventions and health recommendations to pregnant women are essential elements in the prenatal care in Greenland, for example, by improving the maternal food intake and to reduce maternal exposure to alcohol, smoking, cannabis and environmental contaminants (9,10). The recent decade's lifestyle and food transitions are of concern for Greenlandic pregnant women, due to the impact on the developing foetus, birth outcome, and the health of the mother and child (2,11,12).

To our knowledge, this is the very first geographical study of Greenlandic pregnant women for a comprehensive evaluation of lifestyle, reproductive factors and food intake, and possible age and geographical differences.

## Methods and materials

### Study population

**Fig. 1:**
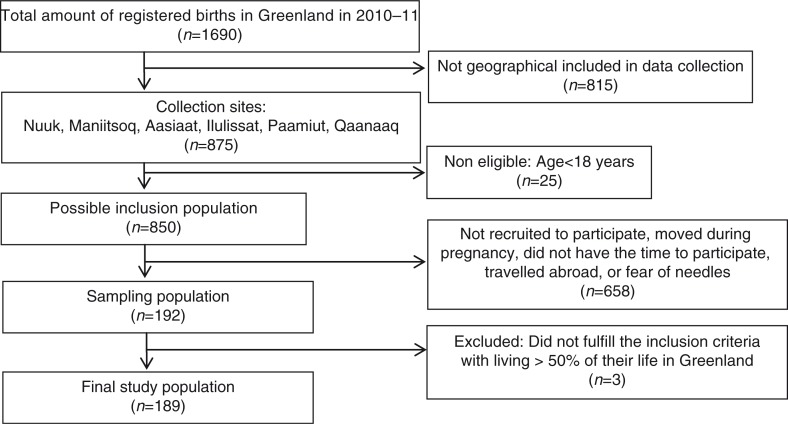
Flow diagram of included Greenlandic pregnant women in the ACCEPT – sub-study, 2010–2011. The participant percent of the total birth in Greenland 2010–2011 was 11.2%. The participant percent of the total birth in the ACCEPT substudy collection sites 2010–2011 was 22.2%. (Source: www.stat.gl).

**Fig. 2:**
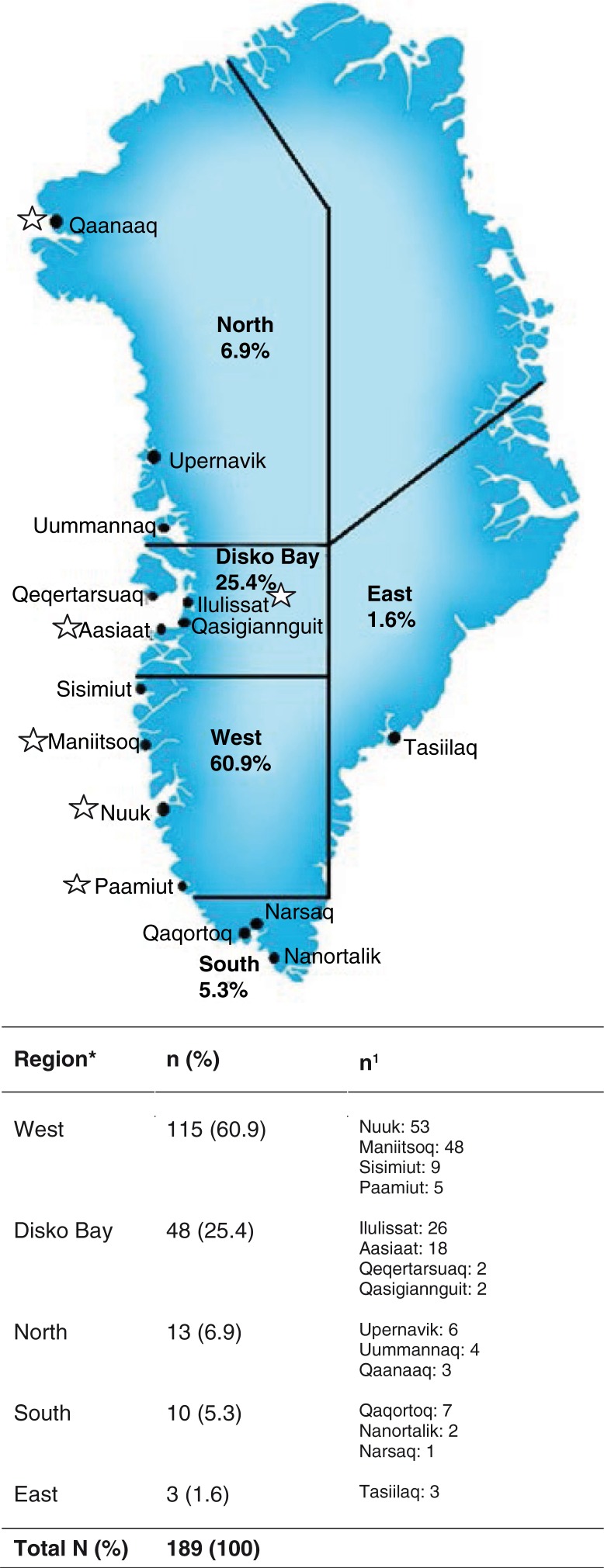
Map of Greenland with the defined regions: West, Disko Bay, South, North, East. * denotes region where participants had lived >50% of their life. N (%): total number of participants; n (%): number of participants in the related regions; n^1^: number of participants distributed in the different towns for each region, respectively. Collection sites are marked with a star.

The overall aim of the ACCEPT (*A*daption to *C*limate *C*hange, *E*nvironmental *P*ollution, and Dietary *T*ransition) was establishment of a geographical and prospective Greenlandic Birth cohort of approximately 600 mother–child pairs including a Biobank (2010–2015). The specific objectives of the ACCEPT project were (a) to evaluate lifestyle, reproductive factors and food intake and (b) to investigate the effects of exposure to environmental POPs on maternal and child health. The present ACCEPT – sub-study includes 189 pregnant women enrolled from August 2010 to September 2011 (Fig. 1). Data collection was conducted in 6 towns (Nuuk, Maniitsoq, Paamiut, Ilulissat, Aasiaat, Qaanaaq) at the Greenlandic west coast. A Danish/Greenlandic-speaking doctor recruited the participants. With the exception from Nuuk, enrollment depended on coast visit carried out by the doctor. Thus, a convenient sampling method was used to cover different geographical parts of Greenland. The inclusion criteria were ≥18 years of age and living >50% of their life in Greenland. The women belonged to the region (West, Disko Bay, North, South or East) in relation to which town they had lived the longest (Fig. 2).

### Questionnaire data

The participants completed two personal interview-based questionnaires in Danish or Greenlandic at enrollment: a culturally appropriate lifestyle questionnaire (LQ) and a specially designed food frequency questionnaire (FFQ). The overall format of the questionnaires had previously been used in the INUENDO project in Greenland (13). The LQ included questions of information about socio-demographic, intake of alcohol (pre- and during pregnancy), ever used cannabis, reproductive history, breastfeeding plans and planned breastfeeding period. The smoking status, current vs. non-current [approximately asked in gestational week (GW) 12], and anthropometric pre-pregnancy body mass index [BMI calculated as weight (in kilograms)/height^2^ (in metres)] (14) was obtained from the women's pregnancy records. Some lifestyle questions were left unanswered. The FFQ evaluated the food intake at inclusion time and included more than 60 food items, which were merged into main food groups for traditional and imported food, respectively. The FFQ was modified during the inclusion period, resulting in missing answers for 10 food items, which led to exclusion for further statistical analysis (see section Statistical Analysis and Supplementary File 1). Traditional food comprised 7 main food groups: marine mammals, seabirds, fish, shellfish, dried fish, terrestrial species and berries (Supplementary Table 2.1). The same applied for imported food with 7 main food groups: meat products, fast food, sauce, carbohydrate food, vegetables, fruit and “snacks and candy” (Supplementary Table 2.2). The consumption frequencies used in the FFQ were not proportional to the exact amount consumed, since portion size was not asked. For this reason, a food consumption frequency score was calculated (Table I), previously used in other studies (15–17). In the FFQ, 8 consumption frequencies were stated for each food item, ranging from “never” to “several times a day.” Each food item was given a frequency score calculating the time(s) a month the food item was consumed (Table I). The intake of each main food group was given as the summed frequency score of included food items for each participant and then the summed frequency scores for all participants were given as median and minimum–maximum (min–max). For further explanation and food item calculation, see example in Supplementary File 3.

**Table I:** Calculation of food consumption frequency score

Consumption frequency	Frequency score [time(s) a month]
Never	0
Rare (less than 1 time a month)	0.5
1 time a month	1
2–3 times a month	2.5
1 time a week	4.3
2–3–4 times a week	13.0
Every day or almost every day	30.4
Several times a day	45.6

The eight consumption frequencies were given as frequency score calculated as the time(s) in a month the food item was consumed.

### Blood samples

Venous blood samples were collected at inclusion. Plasma cotinine (metabolite of nicotine), determined as a predictor of recent tobacco smoking, was analyzed at Centre for Arctic Health & Unit of Cellular and Molecular Toxicology, Aarhus University. The detection limit was 1 ng/ml. Values <1 ng/ml were included in the statistical analysis as 0.500 ng/ml.

### Statistical analysis

Data was double entered into the validation program EpiData. All statistical analyses were performed with SPSS software (SPSS Inc., Chicago, IL, USA). The statistical significant level was set to p<0.05. Natural logarithmic (ln) transformation improved the normality and homogeneity of variance, and the ln data were used. For continuous variables, independent t-test and one-way ANOVA test were used. When ANOVA showed statistical significance, complementary multiple comparison ad hoc tests were performed. Test for equal variances of variables was performed using Levene's test. The least-significant difference (LSD) test was used if equal variance was shown. Otherwise, Dunnett T3 was used. Pearson's Chi-square test was used to check the difference for categorical variables. The statistical analysis in this study examined the data from 189 participants. However, the number of answers for each participant varied. Concerning missing answers in the FFQ a “multiple imputation” method was used (18,19) (Supplementary File 4). This allowed including participants with incomplete data set in the statistical analysis with >5% of missing values, assuming the missing values to be missed at random. Though, 10 food items (Supplementary File 1) with >15% were excluded since they were not missed at random. “Multiple imputation” created multiple different data sets (m>1) with missing values replaced by random plausible values based on the participant's characteristics in the complete data set. The data sets were analyzed and accounting for the variability of the missing values. Analysis of the data showed similarity regarding median, min–max, mean, standard deviation (SD) and 95% confidence interval (95% CI) after multiple imputation for the food items with >5% missing values (Supplementary File 4).

### Ethics

Informed consent was obtained from each woman prior to inclusion. ACCEPT was approved by the Ethical Committee in Greenland in accordance with the Helsinki declaration II.

## Results

### Lifestyle, reproductive factors and food intake

The lifestyle and reproductive factors for the study population are presented in Table II. Median age was 27 years. Median pre-pregnancy BMI was 24.24 kg/m^2^ and 43.3% had BMI>25 kg/m^2^. In the beginning of the pregnancy (approximately in GW 12) 46.3% were current smokers. The current smokers had significantly higher median plasma cotinine level compared to non-current smokers (75.7 ng/ml vs. 0.5 ng/ml, p<0.0001, data not shown). Prevalence of participants ever used cannabis was 45.7%. One woman declared to have been using cannabis during the last year before her pregnancy. Pre-pregnancy alcohol intake showed that 46.7% drank *<*1 time a month. During pregnancy, the majority (96.8%) consumed alcohol *<*1 time a month. Median GW at the inclusion time was 26. Median values for parity and miscarriages were both 1.00. Fifty percent had 1–2 children and 76.7% had ≥1 miscarriage(s). Regarding breastfeeding plans, 97.8% planned to breastfeed their newborn, while 2.2% had no plans; 21.5% planned to breastfeed <6 months; 43.6% between 6 and 18 month, and 26.2% had not considered or planned a breastfeeding period. Table III shows the traditional and imported food intake for the study population. For traditional food, the most consumed food group was marine mammals, followed by fish, while the least consumed food groups were dried fish. The most frequently consumed imported food group was fruit, followed by carbohydrate food and “snacks and candy.” The least consumed was fast food. Overall, the median food intake of traditional and imported food was distributed as 18% and 82%, respectively (Table III).

**Table II:** Characteristics of the study population for lifestyle and reproductive factors

Parameters (N=189)	n (%)	Median	Min–max
Age (years)	189 (100)	27.00	18–42
<27	87 (46.0)	23.00	18–26
≥27	102 (54.0)	31.00	27–42
Pre-pregnancy BMI (kg/m^2^) (missing=9)	180 (100)	24.24	16.38–46.57
<18.49(underweight)	7 (3.9)	17.68	16.38–18.44
18.50–24.99(normal weight)	95 (52.8)	22.35	18.82–24.84
25.00–29.99(overweight)	53 (29.4)	26.91	25.00–29.71
>30.00(obesity)	25 (13.9)	32.69	30.02–46.57
P-cotinine (ng/ml)	189 (100)	0.500	0.500–190.5
Smoking status (missing=1)	188 (100)		
Current	87 (46.3)		
Non-current	101 (53.7)		
Ever used cannabis (missing=14)	175 (100)		
Yes	80 (45.7)		
No	95 (54.3)		
Alcohol intake			
Pre-pregnancy (missing=5)	184 (100)		
<1 time a month	86 (46.7)		
1 time a month	32 (17.4)		
2–3 times a month	37 (20.1)		
>1 time a week	29 (15.8)		
During pregnancy (missing=4)	185 (100)		
<1 time a month	179 (96.8)		
1 time a month	5 (2.7)		
2–3 times a month	1 (0.5)		
>1 time a week	0 (0.0)		
Gestational week (GW) at inclusion	189 (100)	26.00	7–40
1st Trimester (≤12 weeks)	5 (2.6)		
2nd Trimester (13–28 weeks)	109 (57.7)		
3rd Trimester (≥29 weeks)	75 (39.7)		
Parity^a^	189 (100)	1.00	0–6
0	69 (36.5)		
1–2	94 (49.7)		
≥3	26 (13.8)		
Miscarriages^b^	189 (100)	1.00	0–11
0	44 (23.3)		
1–2	100 (52.9)		
≥3	45 (23.8)		
Breastfeeding plans^c^ (missing=4)	185 (100)		
Yes	181 (97.8)		
No	4 (2.2)		
Planned breastfeeding period^c^ (missing=17)	172 (100)		
<6 months	37 (21.5)		
6–12 months	46 (26.7)		
12–18 months	29 (16.9)		
>18 months	15 (8.7)		
Do not know	45 (26.2)		

N: total number of participants in the study population. n: number of participants with answer to the corresponding parameter. Missing: deviation between N and n. Missing answers are not included in the percent calculation (%).

a Number of full-term pregnancies.

b Including spontaneous, medical and surgical miscarriages.

c Breastfeeding question: “Do you plan to breastfeed your child? If yes, for how long?”

**Table III:** The study population intake of traditional and imported food given as time(s) a month for the main food groups

Parameters (N=188)	Median^a^	Min–max^a^
Traditional food intake [time(s) a month] (18%)^b^		
Marine mammals^c^	9.5	0–134.6
Seabirds	1.0	0–32.9
Fish	7.5	0–76.9
Shellfish	3.0	0–31.4
Dried fish	0.5	0–30.4
Terrestrial species^d^	5.0	0–61.8
Berries	1.0	0–30.4
Imported food intake [time(s) a month] (82%)^b^		
Meat products	14.0	1.5–122.1
Fast food	4.5	0–39.0
Sauce	13.0	0–45.6
Carbohydrate food	26.0	0–76.0
Vegetables	13.0	0–45.6
Fruit	31.4	0–167.2
Snacks and candy	22.2	1.5–156.3

N: total number of participants (1 participant was missing the FFQ).

a Median and min–max (see Method – *Questionnaire data*, Table I and Supplementary File 3).

b The overall percentages of median intake of the main food groups, traditional (x) or imported food (y), were calculated by summing the medians of the main food groups and then the sum was divided by the total median intake (x+y).

c Marine mammals: seal, whale, dried meats, blubber.

d Terrestrial species: caribou, muskox, Greenlandic lamb, ptarmigan (see Supplementary File 2).

### Age comparisons

The participants were classified into two comparable age study groups using the median age: <27 years vs. ≥27 years. Table IV shows the comparison of lifestyle and reproductive factors between the age groups. A significant higher rate of parity and miscarriages for women ≥27 years was found. Women <27 years were significantly more in doubt about planned breastfeeding period, 41.0% vs. 13.8% for <27 and ≥27 years, respectively. A trend during pregnancy to consume alcohol once a month more frequently was found for women ≥27 years. Table V shows comparison of traditional and imported food intake for the two age groups. A significant higher consumption of dried fish and fast food was observed for women <27 years. The two age groups had different distribution regarding percentage of median food intake for traditional and imported food, with 20% and 80% for women <27 years, and 17% and 83% for women ≥27 years, respectively (Table V).

**Table IV:** Age comparisons for lifestyle and reproductive factors

Parameters (N=189)			Age groups		

			<27 years	≥27 years	
n (%)			87 (46.0)	102 (54.0)	
Age (years): median (min–max)			23.00 (18–26)	31.00 (27–42)	
Anthropometric factors					p
Pre-pregnancy BMI (kg/m^2^) (missing=9)		n	84	96	0.170^a^
		Median (min–max)	23.80 (16.38–46.57)	24.61 (17.48–40.89)	
P-cotinine, smoking, cannabis use and alcohol intake					
P-cotinine (ng/ml)		n	87	102	0.517^a^
		Median (min–max)	0.500 (0.500–160.0)	0.500 (0.500–190.5)	
Smoking status (missing=1)		n (%)	87 (100)	101 (100)	0.422^b^
		Current (%)	43 (49.4)	44 (43.6)	
		Non-current (%)	44 (50.6)	57 (56.4)	
Ever used cannabis (missing=14)		n (%)	79 (100)	96 (100)	0.565^b^
		Yes (%)	38 (48.1)	42 (43.8)	
		No (%)	41 (51.9)	54 (56.2)	
Alcohol	Pre-pregnancy (missing=5)	n (%)	84 (100)	100 (100)	0.587^b^
		<1 time a month	37 (44.0)	49 (49.0)	
		1 time a month	16 (19.0)	16 (16.0)	
		2–3 times a month	15 (18.0)	22 (22.0)	
		>1 time a week	16 (19.0)	13 (13.0)	
	During pregnancy (missing=4)	n (%)	84 (100)	101 (100)	**0.066** b c
		<1 time a month	83 (98.8)	96 (95.0)	
		1 time a month	0 (0.0)	5 (5.0)	
		2–3 times a month	1 (1.2)	0 (0.0)	
		>1 time a week	0 (0.0)	0 (0.0)	
Reproductive factors					
Gestational week (GW) at inclusion		n	87	102	0.503^a^
		Median (min–max)	27.0 (10–39)	26.0 (7–40)	
Parity^d^		n	87	102	**0.002** a
		Median (min–max)	0.0 (0–3)	1.0 (0–6)	
Miscarriages^e^		n	87	102	**0.001** a
		Median (min–max)	1.0 (0–5)	2.0 (0–11)	
Breastfeeding plans^f^ (missing=4)		n (%)	84 (100)	101 (100)	0.852^b^
		Yes (%)	82 (97.6)	99 (98.0)	
		No (%)	2 (2.4)	2 (2.0)	
Planned breastfeeding period^f^		n=172	78 (100)	94 (100)	**<0.0001** b
(missing=17)		<6 months	12 (15.4)	25 (26.6)	
		6–12 months	21 (26.9)	25 (26.6)	
		>12–18 months	4 (5.1)	25 (26.6)	
		>18 months	9 (11.5)	6 (6.4)	
		Do not know	32 (41.0)	13 (13.8)	

N: total number of participants in the study population. n: number of participants with answer to the corresponding parameter. Missing: deviations between N and n. Missing answers are not included in the percent calculation (%). The bold values indicate significant differences. p-Value calculated with:

a independent t-test and

b Chi-square.

c Borderline significant p-value.

d Number of full-term pregnancies.

e Including spontaneous, medical and surgical miscarriages.

f Breastfeeding question: “Do you plan to breastfeed your child? If yes, for how long?”

**Table V:** Age comparisons for traditional and imported food intake [time(s) a month] in main food groups

Parameters (N=188)	Age groups				

	<27 years		≥27 years		
n (%)	87 (46.0)		102 (54.0)		
Age (years): median (min–max)	23.00 (18–26)		31.00 (27–42)		
	n	Median (min–max)^a^	n	Median (min–max)^a^	p^b^
Traditional food intake [time(s) a month]					
Marine mammals^c^	85	11.3 (0–64.9)	102	8.5 (0–134.6)	0.296
Seabirds	83	1.0 (0–32.9)	99	1.0 (0–26.0)	0.420
Fish	86	7.5 (0–76.9)	101	7.0 (0–53.5)	0.368
Shellfish	86	3.0 (0–31.4)	100	3.0 (0–21.6)	0.490
Dried fish	85	1.0 (0–30.4)	99	0.5 (0–13.0)	**0.001**
Terrestrial species^d^	85	5.0 (0–61.8)	102	5.0 (0.5–39.7)	0.962
Berries	85	2.5 (0–30.4)	98	1.0 (0–30.4)	0.179
Imported food intake [time(s) a month]					
Meat products	86	13.9 (1.5–122.1)	102	14.6 (2.0–93.7)	0.584
Fast food	86	5.5 (0.5–39.0)	101	3.5 (0–26.0)	**0.001**
Sauce	83	13.0 (0.5–45.6)	98	13.0 (0–45.6)	0.855
Carbohydrate food	86	26.0 (3.5–76.0)	101	26.0 (0–76.0)	0.827
Vegetables	85	13.0 (0–45.6)	96	13.0 (0.5–45.6)	0.634
Fruit	86	31.4 (1.5–167.2)	101	31.9 (0–136.8)	0.809
Snacks and candy	86	22.7 (2.5–156.3)	102	22.2 (1.5–135.6)	0.371

N: total number of participants (1 participant was missing the FFQ). n: number of participants with corresponding answer to parameter. The bold values indicate significant differences.

a,c,d See text below Table III. p-Value calculated with:

b independent t-test. Percentage of median intake of the main food groups, traditional (x) or imported food (y), were calculated by summing the medians of the main food groups and then the sum was divided by the total median intake (x+y).

### Region comparisons

Table VI shows the comparison of the regions (West, Disko Bay, North, South and East) for lifestyle and reproductive factors. Statistical significant difference of alcohol intake during pregnancy was found between the regions, where it seemed higher in North, South and West. Participants who said “no” to breastfeeding plans belonged to the regions in the order of North>Disko Bay>West, causing statistical differences between regions. The participants in East had a trend to be slightly older and to have higher median pre-pregnancy BMI. Moreover, a trend towards less smoking in South and East was observed. Table VII shows the comparison of traditional and imported food intake for the regions. Women in Disko Bay consumed significantly less terrestrial species compared to the other regions. The regional order for highest percentage of traditional food intake was North>West≥East>South>Disko Bay. A trend to lower intake of berries was found for Disko Bay, and a trend to lower fruit intake was observed for North, compared to other regions (Table VII).

**Table VI:** Region comparisons for lifestyle and reproductive factors

Parameters (N=189)			Region^a^					

			West	Disko Bay	North	South	East	
n (%)			115 (60.9)	48 (25.4)	13 (6.9)	10 (5.3)	3 (1.6)	p
Age (years): median (min–max)			27.00 (18–42)	27.00 (19–40)	27.00 (21–35)	27.50 (22–41)	32.00 (32–37)	0.231^b^
Anthropometric factors								
Pre-pregnancy BMI (kg/m^2^) (missing=9)		n	109	47	11	10	3	0.330^b^
		Median (min–max)	24.61 (16.38–46.57)	24.44 (17.54–39.33)	23.53 (20.06–29.05)	22.34 (20.55–24.84)	28.91 (23.73–29.71)	
P-cotinine, smoking, cannabis use and alcohol intake								
P-cotinine (ng/ml)		n	115	48	13	10	3	0.260^b^
		Median (min–max)	0.500 (0.500–190.5)	6.94 (0.500–190.5)	15.17 (0.500–60.0)	0.500 (0.500–126.1)	0.500 (0.500–7.5)	
Smoking status (missing=1)		n (%)	115 (100)	48 (100)	12 (100)	10 (100)	3 (100)	**0.085** c d
		Current (%)	49 (42.6)	27 (56.2)	8 (66.7)	3 (30.0)	0 (0.0)	
		Non-current (%)	66 (57.4)	21 (43.8)	4 (33.3)	7 (70.0)	3 (100)	
Ever used cannabis (missing=14)		n (%)	106 (100)	45 (100)	11 (100)	10 (100)	3 (100)	0.410^c^
		Yes (%)	50 (47.2)	21 (46.7)	6 (54.5)	3 (30.0)	0 (0.0)	
		No (%)	56 (52.8)	24 (53.3)	5 (45.5)	7 (70.0)	3 (100)	
Alcohol	Pre-pregnancy (missing=5)	n (%)	113 (100)	45 (100)	13 (100)	10 (100)	3 (100)	0.192^c^
		<1 time a month	53 (46.9)	20 (44.4)	4 (30.8)	7 (70.0)	2 (66.7)	
		1 time a month	25 (22.1)	6 (13.3)	1 (7.7)	0 (0.0)	0 (0.0)	
		2–3 times a month	21 (18.6)	8 (17.8)	6 (46.2)	1 (10.0)	1 (33.3)	
		>1 time a week	14 (12.4)	11 (24.4)	2 (15.4)	2 (2.0)	0 (0.0)	
	During pregnancy (missing=4)	n (%)	114 (100)	46 (100)	13 (100)	9 (100)	3 (100)	**0.018** c
		<1 time a month	111 (97.4)	46 (100)	11 (84.6)	8 (88.9)	3 (100)	
		1 time a month	3 (2.6)	0 (0.0)	1 (7.7)	1 (11.1)	0 (0.0)	
		2–3 times a month	0 (0.0)	0 (0.0)	1 (7.7)	0 (0.0)	0 (0.0)	
		>1 time a week	0 (0.0)	0 (0.0)	0 (0.0)	0 (0.0)	0 (0.0)	
Reproductive factors								
Gestational week at inclusion		n=189	115	48	13	10	3	0.507^b^
		Median (min–max)	26.0 (10–40)	26.5 (13–40)	27.0 (7–38)	27.0 (16–39)	33.0 (24–34)	
Parity^e^		n=189	115	48	13	10	3	0.165^b^
		Median (min–max)	1.0 (0–6)	1.0 (0–4)	1.0 (0–5)	1.0 (0–2)	2.0 (2–3)	
Miscarriages^f^		n=189	115	48	13	10	3	0.233^b^
		Median (min–max)	1.0 (0–9)	2.0 (0–11)	1.0 (0–4)	0.0 (0–4)	2.0 (1–4)	
Breastfeeding plans^g^ (Missing=4)		n (%)	113 (100)	47 (100)	12 (100)	10 (100)	3 (100)	**0.011** c
		Yes (%)	112 (99.1)	46 (97.9)	10 (83.3)	10 (100)	3 (100)	
		No (%)	1 (0.9)	1 (2.1)	2 (16.7)	0 (0.0)	0 (0.0)	
Planned breastfeeding period^g^		n (%)	103 (100)	46 (100)	10 (100)	10 (100)	3 (100)	0.675^c^
(missing=17)		<6 months	19 (18.4)	13 (28.3)	2 (20.0)	3 (30.0)	0 (0.0)	
		6–12 months	29 (28.2)	10 (21.7)	3 (30.0)	4 (40.0)	0 (0.0)	
		>12–18 months	17 (16.5)	8 (17.4)	2 (20.0)	1 (10.0)	1 (33.3)	
		>18 months	12 (11.7)	1 (2.2)	0 (0.0)	1 (10.0)	1 (33.3)	
		Do not know	26 (25.2)	14 (30.4)	3 (30.0)	1 (10.0)	1 (33.3)	

N: total number of participants in the study population. n: number of participants with answer to the corresponding parameter. Missing: deviations between N and n. Missing answers are not included in the percent calculation (%). The bold values indicate significant differences.

a Region where the participants had lived >50% of their life. p-Value calculated with:

b one-way ANOVA

c Chi-square test.

d Borderline significant p-value.

e Number of full-term pregnancies.

f Including spontaneous, medical and surgical miscarriages.

g Breastfeeding question: “Do you plan to breastfeed your child? If yes, for how long?”

**Table VII:** Region comparisons for traditional and imported food intake [time(s) a month] in main food groups

Parameters (N=188)	Region^a^										

	West		Disko Bay		North		South		East		
n (%)	115 (60.9)		48 (25.4)		13 (6.9)		10 (5.3)		3 (1.6)		
Age (years): median (min–max)	27.00 (18–42)		27.00 (19–40)		27.00 (21–35)		27.50 (22–41)		32.00 (32–37)		
	n	Median (min–max)^b^	n	Median (min–max)^b^	n	Median (min–max)^b^	n	Median (min–max)^b^	n	Median (min–max)^b^	p^c^
Traditional food intake [time(s) a month]											
Marine mammals^d^	114	8.3 (0–64.9)	48	9.8 (2.0–134.6)	13	14.0 (3.5–36.1)	9	9.0 (5.5–29.4)	3	14.0 (11.5–49.3)	0.126
Seabirds	114	1.0 (0–26.0)	44	0.5 (0–32.9)	12	0.8 (0–5.0)	9	1.0 (0.5–3.0)	3	1.0 (1.0–2.0)	0.287
Fish	114	7.5 (0.5–76.9)	48	5.8 (0–52.1)	13	8.0 (1.5–31.2)	9	7.5 (2.0–29.8)	3	13.5 (8.0–41.9)	0.166
Shellfish	114	3.0 (0–31.4)	47	2.5 (0–31.4)	13	1.5 (0–16.0)	9	4.0 (1.0–18.0)	3	5.5 (3.5–21.6)	0.136
Dried fish	113	0.5 (0–30.4)	46	0.5 (0–30.4)	13	1.0 (0–2.50)	9	1.0 (0.5–4.3)	3	0.5 (0.5–4.3)	0.579
Terrestrial species^e^	115	5.5 (0–61.8)	47	3.0 (0.5–30.4)	13	5.8 (0.5–13.4)	9	6.0 (0–10.0)	3	6.0 (3.5–20.8)	**0.002**
Berries	114	2.5 (0–30.4)	44	0.5 (0.5–30.4)	13	2.5 (0–30.4)	9	2.5 (1–30.4)	3	2.5 (2.5–13.0)	**0.082** f
Imported food intake [time(s) a month]											
Meat products	115	13.9 (2.5–122.1)	48	13.7 (2.0–65.1)	13	19.5 (3.0–69.9)	9	12.1 (1.5–42.0)	3	35.1 (11.0–47.6)	0.508
Fast food	115	5.3 (0.5–39.0)	47	4.5 (0–26.0)	13	3.5 (1.0–9.3)	9	3.5 (1.5–30.3)	3	5.8 (2.5–7.5)	0.665
Sauce	110	13.0 (0–45.6)	46	21.7 (2.5–45.6)	13	30.4 (2.5–30.4)	9	13.0 (0.5–30.4)	3	30.4 (30.4–30.4)	0.461
Carbohydrate food	115	26.0 (1.0–60.8)	47	43.4 (0–76.0)	13	26.0 (5.0–76.0)	9	26.0 (3.0–60.8)	3	34.7 (26.0–43.4)	0.170
Vegetables	115	13.0 (0.5–45.6)	46	13.0 (0–45.6)	10	13.0 (0.5–30.4)	8	30.4 (2.5–45.6)	2	38.0 (30.4–45.6)	0.423
Fruit	115	31.3 (0.5–149.8)	47	32.9 (0.5–167.2)	13	11.9 (0–47.1)	9	45.4 (20.5–104.7)	3	35.9 (2.0–89.5)	0.163
Snacks and candy	115	23.3 (2.5–156.3)	48	5.8 (2.0–121.5)	13	20.4 (1.5–69.3)	9	35.6 (16.8–130.2)	3	25.8 (8.5–104.1)	0.309

N: total number of participants (1 participant was missing the FFQ). n: number of participants with corresponding answer to parameter. The bold values indicate significant differences.

a Region where the participants had lived >50% of their life.

b,d,e See text below Table III. p-Value calculated with.

c one-way ANOVA test.

f Borderline significant p-value. Percentage of median intake of the main food groups, traditional (x) or imported food (y), were calculated by summing the medians of the main food groups and then the sum was divided by the total median intake (x+y).

### Trimester stratifying

Supplementary File 5 shows the comparison of lifestyle behaviour parameters (p-cotinine, smoking status, ever used cannabis, alcohol intake during pregnancy, breastfeeding plan and planned breastfeeding period) for the two age groups (<27 years vs. ≥27 years) and for the regions groups (West, Disko Bay, North, South, East) stratifying by trimester. The percent distribution was 2.6, 57.7 and 39.7% in 1st, 2nd and 3rd trimester, respectively. Because of the very few participants in 1st trimester the statistical data of 1st trimester might be chance findings. For the age groups (Supplementary Table 5.1), a significant difference was seen regarding planned breastfeeding period both in 2nd and 3rd trimester, since more women <27 years were in doubt, as found for the analyses of all data. However, in contrast to the pooled data, no trend was seen regarding alcohol intake during pregnancy when comparing age groups stratified by trimester. For the region groups (Supplementary Table 5.2), a significant difference was seen regarding alcohol intake during pregnancy in 2nd trimester, since it seemed higher in North. The breastfeeding plans also showed significant difference in 2nd trimester, since pregnant women in North had the highest percent of “no” answers. No trend was seen regarding smoking status when comparing region groups stratifying by trimester.

## Discussion

The present ACCEPT – sub-study provides data for lifestyle, reproductive factors and food intake during 2010–2011 for 189 Greenlandic pregnant women, and found some age and geographical differences. Pregnancy is an essential period in life to make fundamental changes. Lifestyle, reproductive factors and food intake can affect the intrauterine environment being vital for the developing foetus. Pregnancy in Greenland is challenged by several factors, which, to some extent, may not be considered in the National Southern counterparts, such as Denmark. Critical factors that can influence pregnancy are, for example, diverse prenatal care, great turnovers among the health staff, high exposure to environmental contaminants via the marine food web, women with risk pregnancies are moved from their families to the main hospital, Queen Ingrid Hospital, in Nuuk. In addition, the rapid transitions of cultural and social aspects in the Arctic within few decades also have impact on maternal health. In accordance with the WHO recommendation, it is essential to continue improving maternal health in Greenland, and enable the women to go safely through pregnancy and childbirth, and provide the best opportunities for having a healthy child (20).

The population characteristics showed that 43.3% had a pre-pregnancy BMI >25.0 kg/m^2^ and 46.3% were current smokers in the beginning of their pregnancy. Both findings are of concern and can affect the maternal and child health status. The gestational week at inclusion ranged from 7 to 40 (median GW 26), which may affect the time to have considered a breastfeeding plan. We observed that alcohol intake was reduced when pregnancy was discovered, indicating a healthy trend. The food intake, compared to former studies (5,21–24), showed a relatively similar, though slightly lower, intake of traditional vs. imported food, 18% and 82%, respectively.

Lifestyle factors showed a trend to larger alcohol intake during pregnancy in the age group ≥27 years, which might be a leftover of previous habits compared to newer alcohol recommendations (25). As expected, the age group <27 years had significantly lower parity, fewer miscarriages, had a shorter planned breastfeeding period, and were more in doubt about planned breastfeeding period, compared to the age group ≥27 years. After stratifying by trimester, the age difference for women in the 2nd and 3rd trimesters was similar to the whole population, which was expected since 97.4% of women were in the 2nd and 3rd trimesters at inclusion. We found a significant difference between the age groups, regarding food intake of dried fish and fast food; women <27 years of age had higher intake of both. Previous studies have reported that the young population in Greenland depends more on imported food items (1,2,5). We confirmed that the women of <27 years of age rely more on Westernized food items, such as pizza, food from a grill bar and prepared meals. These food items are of great concern for the public health, since they are high in trans-fatty acids and can cause obesity, high blood pressure, and thereby cardiovascular diseases (CVD) and diabetes mellitus type 2 (DM2) (26).

The 5 regions were defined as where the women had lived the longest time of their lives, presumed to reflect cultural- and geographical-related lifestyle, reproductive factors and food intake. Behaviours achieved in childhood and youth are often brought with later in life. The present ACCEPT – sub-study is geographically representative for pregnant women primarily living longest in the central west Greenland. For regional differences, we found that the women from North, South and West, had a slightly higher alcohol intake during pregnancy. This was also found by stratifying by trimester, where a significant difference in 2nd trimester was found, since 2nd trimester dominated the data. The breastfeeding plans were significantly lower for women in the North, which can be explained by two of this region's participants who planned to give their child up for adoption. Hence, the regional differences were similar to the whole population when stratifying by trimester. Women from Disko Bay consumed significantly less terrestrial species, which among others is due to the limited herds of reindeer in the Disko Bay area (27). Women from the North had a trend to consume less fruit, reflecting the challenged supply opportunities, required extended shelf-time, and changing weather conditions in these distant areas of Greenland. The difference in fruit intake in North was significantly lower, when comparing with South. Consequently, pregnant women in Greenland have different opportunities to consume healthy food items, such as fruit, depending on their geographical residence. No significant differences between regions were found for intake of marine mammals or seabirds.

The present ACCEPT – sub-study can be compared with previous Greenlandic Birth cohorts (Table VIII). The cohorts include a varied number of participants, but to some extent with overlapping collection sites. Age data for the present study are comparable with the other Greenlandic Birth cohorts. For the BMI, Inuit body proportions may be evaluated, with regard to torso proportion and abdominal circumference, muscle mass and fat distribution, which differs from Caucasians (28,29). A previous Greenlandic study reported that standardized WHO cut-off for BMI above 25 kg/m^2^ was not comparable for Inuit's, and may overestimate the number of individuals with high BMI (29). The Disko Bay Birth Cohort (DBBC) (17) had a relatively lower mean pre-pregnancy BMI, compared to pregnant women from the Disko Bay region in our present study (Table VIII). Thus, 10% temporal increase in mean BMI for women in the Disko Bay area from 1994–1996 to 2010–2011 was found. In addition, pre-pregnancy BMI in the present study was comparable with the pre-pregnancy BMI from the INUENDO Cohort (2002–2004) (13) (Table VIII). The health aspects of mothers and children in Greenland are of concern, since overweight/obesity rates in Greenland are increasing (23,30), and especially, the comorbid conditions, such as DM2, CVD and metabolic syndrome, can affect future generations through foetal programming. Based on the present study, we recommend monitoring of obesity rates of pregnant women in Greenland to establish relevant intervention programs. The alcohol questions in the present study were fairly vague and an option for alcohol consumption “0 time a month” was absent in the questionnaire. Alcohol consumption during pregnancy was not a problem, since only a few participants consumed alcohol during pregnancy. These few women might have been recommended to stop alcohol intake, for example, by consulting a doctor or midwife. The IVAAQ cohort (31) reported that the majority were abstainers with 0 drinks per week during pregnancy (Table VIII), which overall was comparable with our findings. Prenatal alcohol exposure has been connected to foetal alcohol syndrome with growth deficiency, craniofacial abnormalities and mental retardation (1,32). An intense awareness of pregnant women, who are drinking alcohol during pregnancy, is highly recommended in Greenland.

**Table VIII:** Comparison of population characteristics for the ACCEPT – sub-study with previous Greenlandic Birth cohorts

Parameters	ACCEPT – sub-study	Disko Bay Birth Cohort (DBBC) (17)	INUENDO (13)	IVAAQ cohort (31)
Year	2010–2011	1994–1996	2002–2004	1999–2005
Participants (n)	189	135	525	403
Collection sites	Nuuk, Maniitsoq, Ilulissat, Aasiaat, Paamiut, Qaanaaq	Ilulissat, Aasiaat, Qasigiannguit, Qeqertarsuaq	Aasiaat, Ilulissat, Kangaatsiaq, Maniitsoq, Nanortalik, Narsaq, Nuuk, Paamiut, Qaanaaq, Qaqortoq, Qasigiannguit, Qeqertarsuaq, Sisimiut, Uummannaq, Tasiilaq, Kulusuk, Kuummiut, Saattut, Ukkusissat	Nuuk, Maniitsoq, Ilulissat
Lifestyle factors				
Age (years): median (min–max)	27.0 (18–42)	27.0 (16–42)	26.0 (20–36)	27.4^a^ (16–46)
Pre-pregnancy BMI (kg/m^2^): median	24.24 25.31^a^ (Disko Bay region)	22.85^a^	24.0	na
No alcohol during pregnancy	96.8%^b^	na	na	92.4%^c^
Current smokers	46.3% 56.2% (Disko Bay region)	60%	56%	45%
Food intake				
Traditional food intake	Comparison of ACCEPT – sub-study and DBBC: Pregnant women in the Disko Bay region in the present ACCEPT – sub-study consumed less seals and seabirds, and more terrestrial species^d^, compared to the pregnant women in the DBBC.		na	na

n=amount of participants. na=not asked/not available.

a Mean value.

b Alcohol intake <1 time a month.

c Alcohol intake 0 drinks of alcohol per week.

d Sum of caribou, hare and muskox.

The smoking status in the present study was obtained from the pregnancy records, which reported current or non-current smokers. Hence, the smoking question was incomplete and not suitable for analysis of pack years, since the numbers of cigarettes smoked daily and the amount of smoking years was absent. Significant higher plasma cotinine level among current smokers, compared to non-smokers, confirmed the actual smoking status. In the present study and for previous Greenlandic Birth cohorts (Table VIII), the percentage of current smokers is high (range 45–60%), which is of high concern indicating no temporal decrease. Midwives and doctors in Greenland encourage pregnant women to quit smoking at their first prenatal visit (approximately at GW 12), and if they are smokers to be offered a smoking cessation. Whether this was conducted in the present study is unknown. The number of current smokers in our study was similar to the smokers in the IVAAQ cohort (31) and slightly smaller than found in the DBBC (17) and Inuendo (13) cohort studies (Table VIII). Hopefully, the smoking was reduced during pregnancy for the ACCEPT women in our study as found for IVAAQ cohort (31), where 25% stated to have stopped smoking during pregnancy. Previous studies have shown that prenatal exposure to smoking can cause spontaneous abortion, intrauterine growth retardation, sudden infant death syndrome and low birth weight (1,32). The high smoking rates during pregnancy are of great concern in Greenland, and a recommendation action should be introduced.

The FFQ evaluated food intake at inclusion time and was used to get an impression of the participant's food consumption at enrollment. The related food habits are associated with the region where the pregnant women had lived the longest, which in our study was >50% of their life. One limitation was the missing data in the FFQ, though the method with multiple imputation gave similar data. Another factor, which may influence the food intake, was inclusion at different times of the year, which introduces a seasonal variation in food supply in the different collection sites. The long-term food intake and day-to-day variation is not covered by a single answered FFQ, which may lead to information bias.

**Fig. 3:**
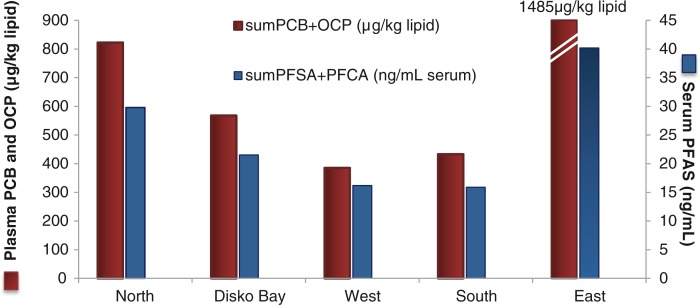
Persistent organic pollutants (POPs) levels in Greenlandic pregnant women in the ACCEPT – sub-study, 2010–2011. Y-axis: the sum of legacy POPs (red bar); polychlorinated biphenyls (PCB) and organochlorine pesticides (OCP) in µg/kg lipid. Z-axis (blue bar): the sum of perfluoroalkylated substances (PFAS); perflourosulfonated acids (PFSA) and perflourocarboxylated acids (PFCA) in ng/ml serum.

In general, we found similar but a slightly lower percent intake of traditional food compared to earlier studies in Greenland (5,23,24). That supports the lowest serum POP level ever found for pregnant women in Greenland (8). As seen before the profile of higher serum POP levels in participants from the North and East were still clear (Fig. 3), which reflects the high percentage of traditional food intake in these 2 regions, although only 3 participants in East (Table VII). A temporal decrease for traditional food intake was seen for pregnant women from the Disko Bay region in the present study, compared to the DBBC (17), since less seals and seabirds were consumed. However, more terrestrial species were consumed in the present study compared to DBBC, supposedly reflecting a shift into a more “traditional-terrestrial” diet and today's import and availability in supermarket (Table VIII). Concerning traditional marine food containing environmental contaminants, the Greenlandic Nutritional Council has published the food-based dietary guidelines (FBDGs) with 10 separate pieces of advice to pregnant and breastfeeding women in Greenland (9). It could be suggested to distribute these FBDGs to the young girls in the Greenlandic public school and to women in the childbearing age, since lipophilic POPs have long half-lives and bioaccumulate in the marine mammals and humans. The lipophilic POPs are found mainly in the food items like blubber and mattak (skin from marine mammals, and for metals like mercury in the liver) which were eaten long before the women become pregnant. By informing the younger generations about the contaminants, in relation to specific traditional marine food items, over time there should be a reduction in POP exposure of women, and thereby the developing foetus and the following generation.

In summary, data on lifestyle, reproductive factors and food intake are presented for 189 Greenlandic pregnant women during 2010–2011, and we found some age and geographical differences. Relevant maternal intervention programs in Greenland are needed, for example, by focussing at obesity risks, smoking cessation, breastfeeding benefits, food recommendations for pregnant women, and promoting the health advantages of some traditional food items, such as terrestrial items and fish (low in POPs). Although, an improvement in lower POP serum levels, a further diet recommendation is important.

## Supplementary Material

Lifestyle, reproductive factors and food intake in Greenlandic pregnant women: The ACCEPT – sub-study
