# Supplementary material for: Lifestyle, reproductive factors and food intake in Greenlandic pregnant women: The ACCEPT – sub-study
Source: Int J Circumpolar Health. 2015 Nov 17;74:10.3402/ijch.v74.29469. doi: 10.3402/ijch.v74.29469 (PMC4652332; doi:10.3402/ijch.v74.29469)
Supplement: Lifestyle, reproductive factors and food intake in Greenlandic pregnant women: The ACCEPT – sub-study [file IJCH-74-29469-s001.pdf]

SUPPLEMENTARY MATERIALS

Lifestyle, reproductive factors and food intake in Greenlandic pregnant women: The ACCEPT - sub study

Submission: International Journal of Circumpolar Health

Supplementary 1: Excluded food items

During the inclusion period the FFQ was modified, resulting in a 1st and 2nd FFQ version (with a Danish and Greenlandic translation), leading to exclusion of 10 food items (*Suppl. table 1*). The explanation is as follows: a mistranslation concerning walrus appeared in the Greenlandic version of the FFQ, both 1st and 2nd version. Some food items (polar bear, guillemot, dried capelin, dried cod, dried catfish, hare, and rice) were missing in the 1st FFQ version. Furthermore, two food items were omitted: 1) "blubber to dried fish", since it was a double-asked food item question, which could be misunderstood; 2) hooded seal, since less than 100 hooded seals have been hunted the last couple of years in Greenland. These food items could not be included in the *Multiple Imputation* method (*Suppl. 4*), because > 15% had missing data, which gives too large statistical variations.

**N = 188**	**1st FFQ version**		**2nd FFQ version**		**Asked (n)**	**Not asked (n)**	**Total (N)**
**Excluded food items**	*Danish: n = 36*	*Greenlandic: n = 31*	*Danish: n = 29*	*Greenlandic: n = 92*			
Walrus	Asked	Mistranslation	Asked	Mistranslation	65	122#	**187#**
Polar bear	Not asked	Not asked	Asked	Asked	121	67	**188**
Guillemot	Asked	Not asked	Asked	Asked	157	31	**188**
Dried capelin	Not asked	Not asked	Asked	Asked	121	67	**188**
Dried cod	Not asked	Not asked	Asked	Asked	121	67	**188**
Dried catfish	Not asked	Not asked	Asked	Asked	121	67	**188**
Hare	Not asked	Not asked	Asked	Asked	121	67	**188**
Rice	Asked	Not asked	Asked	Asked	157	31	**188**
Blubber to dried fish	Double-asked question, which could be misunderstood				Excluded		
Hooded seal	< 100 hunted hooded seals in Greenland the last couple of years *				Excluded		

Suppl. table 1. FFQ versions and 10 excluded food items. The different FFQ versions (1st & 2nd FFQ versions with a Danish and Greenlandic translation) and excluded food items with missing answers and explanations. **N** = total amount of answered FFQ for participants (one participant was missing the FFQ). **n** = number of FFQs and answers in the different versions. # One participant filled in a 1st FFQ version in Danish, and was missing the first page of the FFQ with marine mammals including walrus. * Reference: NANOQ - The Greenlandic Autonomy: Department of Fishing, Hunting and Agriculture. Seal species [in Danish]: Available at: http://naalakkersuisut.gl/~/media/Nanoq/Files/Publications/Fangst%20og%20fiskeri/DK/UdgivelserFJAFactsheet%20GRL%20sealApril2012dk%20DOK896169.pdf

Supplementary 2: Main food groups

Below are stated two supplementary tables showing the specific food items categorized in each main food group. Traditional food (*Supp. table 2.1*) and imported food (*Suppl. table 2.2*) each with seven main food groups and the included food items. The 10 excluded food items in *Supplementary 1* were not a part of the main food groups.

	Main food groups						
	**Marine mammals**	**Seabirds**	**Fish**	**Shellfish**	**Dried fish**	**Terrestrial species**	**Berries**
Food items	*Seal* [1]	*Eider*	*Trout*	*Shrimps*	*Dried Greenlandic halibut*	*Caribou*	*All kinds*
	Whale [2]	*Kittiwake* *	*Cod*	*Clamps* *		*Muskox*	
	Dried meats [3]		*Greenlandic halibut*	*Crab*		*Greenlandic lamb*	
	Blubber		*Atlantic halibut* *			*Ptarmigan* *	
	Seal or whale with rice		*Redfish* *				
			Atlantic salmon *				
			Capelin				

Suppl. table 2.1. Traditional food with the main food groups including the specific food items. [1] *Seal*: All seal species, excluding hooded seal. [2] *Whale*: minke whale, fin whale, beluga whale, narwhale, and harbour porpoise/grind. [3] *Dried meats:* from minke whale, fin whale, beluga whale, narwhale or seal. * Missing answers > 5%, where multiple imputation was used, see *Suppl. table 4.*

	Main food groups						
	**Meat products**	**Fast food**	**Sauce**	**Carbohydrate food**	**Vegetables**	**Fruit**	**Snacks & candy**
Food items	*Chicken / turkey*	*Pizza*	*All kinds*	*Potatoes*	*All kinds*	*Fresh*	*Cake*
	Pork	*Prepared meals*		*Pasta*		*Canned*	*Nuts incl. peanuts*
	Beef	*Food from grill bar*				*Frozen*	*Chocolate (milk)*
	Imported lamb					*Dried*	*Chocolate (dark)*
	Imported fish *						*Candy*
							Chips

Suppl. table 2.2. Imported food with the main food groups including the specific food items. ***** Missing answers > 5%, where multiple imputation was used, see *Suppl. table 4.*

Supplementary 3: Food intake calculation

Example: Food intake of "Seabirds" for the study population

For traditional food, the food intake for the main food group (here Seabirds) was given as a median frequency score by summing the frequency scores of included food items (here 'Eider' and 'Kittiwake'), which gave a median intake of food consumption (time(s) a month) of Seabirds.

Calculation example: the intake of Seabirds (time(s) a month) for the study population:

Participant	Eider	Kittiwake	Sum Seabirds = (Eider + Kittiwake)
1	Never = 0	Never = 0	0.0 times a month
2	2 -3 times a month = 2.5	Every day or almost every day = 30.4	32.9 times a month
3	1 time a week = 4.3	1 time a week = 4.3	8.6 times a month
4	1 time a month = 1	2 -3 times a month = 2.5	3.5 times a month
5	1 time a week = 4.3	Rare (less than 1 time a month) = 0.5	4.8 times a month
…	…	…	…
…	…	…	…
188	Rare (less than one time a month) = 0.5	Never = 0	0.5 times a month

These calculations for food intake regarding 'Seabirds' were made for the whole study population (N = 188, since one was missing the FFQ). The intake of each main food group was given as a the summed frequency score of included food items for each participant and then the summed frequency scores for all participants were given as Median and Min-Max. In summary, the study population (N = 188) consumed 'Seabirds' 1.0 time(s) a month and with a Min – Max: 0 – 32.9 time(s) a month (see *Table 3*).

Hence, these calculations were completed for all main food groups (*Suppl. 2.*) in the study population (*Table 3*), and for the age (*Table 5*) and regions groups (*Table 7*).

10 food items, which had > 15% missing data were excluded from the food intake calculations (*Suppl. 1*). Some food items had missing answers > 5% but < 15%, which resulted in using the *Multiple Imputation* method (*Suppl. 4* and see text in **Methods** **-** *Statistical analysis* for further explanation).

Supplementary 4: Multiple imputation

Parameter	Median	Min-Max	Mean	SD	95% CI
Seabirds: Sum of Eider, Kittiwake *					
Original dataset	1.0	0 – 32.9	1.6	3.3	1.1 – 2.1
1 imputation dataset	1.0	0 – 32.9	1.7	3.3	1.2 – 2.2
2 imputation dataset	1.0	0 – 32.9	1.6	3.3	1.2 – 2.1
3 imputation dataset	1.0	0 – 32.9	1.6	3.3	1.2 – 2.1
4 imputation dataset	1.0	0 – 32.9	1.7	3.4	1.2 – 2.2
5 imputation dataset	1.0	0 – 32.9	1.7	3.3	1.2 – 2.1
Fish: Sum of Trout, Cod, Greenlandic Halibut, Atlantic Halibut *, Redfish *, Atlantic salmon *, Capelin					
Original dataset	7.5	0 – 76.9	10.1	11.1	8.5 – 11.7
1 imputation dataset	7.5	0 – 76.9	10.3	11.1	8.7 – 11.9
2 imputation dataset	7.5	0 – 76.9	10.2	11.1	8.6 – 11.8
3 imputation dataset	7.5	0 – 76.9	10.2	11.1	8.6 – 11.8
4 imputation dataset	7.5	0 – 76.9	10.3	11.1	8.7 – 11.9
5 imputation dataset	7.5	0 – 76.9	10.2	11.1	8.7 – 11.8
Shellfish: Sum of Shrimps, Clamps*, Crab					
Original dataset	3.0	0 – 31.4	3.9	4.7	3.2 – 4.6
1 imputation dataset	3.0	0 – 32.9	3.9	4.7	3.2 – 4.6
2 imputation dataset	3.0	0 – 34.8	3.9	4.8	3.2 – 4.6
3 imputation dataset	3.0	0 – 33.5	3.9	4.7	3.3 – 4.6
4 imputation dataset	3.0	0 – 36.0	3.9	4.8	3.2 – 4.6
5 imputation dataset	3.0	0 – 31.7	3.9	4.7	3.2 – 4.6
Terrestrial species: Sum of Caribou, Muskox, Greenlandic lamb, Ptarmigan*					
Original dataset	5.0	0 – 61.8	6.9	7.8	5.7 – 8.0
1 imputation dataset	5.0	0 – 61.8	6.9	7.8	5.7 – 8.0
2 imputation dataset	5.0	0 – 61.8	6.9	7.8	5.7 – 8.0
3 imputation dataset	5.0	0 – 61.8	6.9	7.8	5.7 – 8.0
4 imputation dataset	5.0	0 – 61.8	6.9	7.8	5.7 – 8.0
5 imputation dataset	5.0	0 – 61.8	6.9	7.8	5.7 – 8.0
Meat products: Sum of Chicken / Turkey, Pork, Beef, Imported Lamb, Imported fish *					
Original dataset	14.0	1.5 – 122.1	20.5	17.9	18.0 – 23.1
1 imputation dataset	14.1	1.5 – 122.1	20.8	18.1	18.2 – 23.4
2 imputation dataset	14.1	1.5 – 122.1	20.7	18.0	18.2 – 23.3
3 imputation dataset	14.1	1.5 – 122.1	20.6	17.9	18.1 – 23.2
4 imputation dataset	14.1	1.5 – 122.1	20.7	18.0	18.1 – 23.3
5 imputation dataset	14.2	1.5 – 122.1	20.8	17.9	18.2 – 23.4

Suppl. table 4. Multiple imputation for 7 food items with > 5% missing answers included in main food groups. * : imputed food items. The 7 imputed food items were summed with the other food items belonging to the corresponding main food groups (*Suppl. 2*). SD = Standard deviation. CI = confidence interval. See text in **Methods** - *Statistical analysis* for further explanation.

Supplementary 5: Trimester stratifying

**Parameter**		**1st trimester**		**2nd trimester**		**3rd trimester**	
		< 27 years	≥ 27 years	< 27 years	≥ 27 years	< 27 years	≥ 27 years
P-cotinine (ng/ml)	*n*	*2*	*3*	*48*	*61*	*37*	*38*
	Median	0.5	46.7	0.5	0.5	1.0	0.5
	Min-Max	0.5-0.5	0.5-52.8	0.5-160	0.5-190	0.5-126	0.5-190
	p-value	*0.7*		*0.53*		*0.22*	
Smoking status	*n*	*2*	*3*	*48*	*61*	*37*	*37*
	Current(%)	50%	66.7%	41.7%	41%	59.5%	45.9%
	Non-current(%)	50%	33.3%	58.3%	59%	40.5%	54.1%
	p-value	*0.71*		*0.94*		*0.24*	
Ever used cannabis	*n*	*2*	*3*	*46*	*58*	*31*	*35*
	Yes	50%	100%	41.3%	37.9%	58.1%	48.6%
	No	50%	0	58.7%	62.1%	41.9%	51.4%
	p-value	*0.40*		*0.73*		*0.44*	
Alcohol	*n*	*2*	*3*	*48*	*61*	*34*	*37*
During pregnancy	<1 time/month(%)	100%	100%	97.9%	93.4%	100%	97.3%
	1 time/month(%)	0	0	0	6.6%	0	2.7%
	2-3 time/month(%)	0	0	2.1%	0	0	0
	>1 time/week(%)	0	0	0	0	0	0
	p-value	*ns*		*0.11*		*0.33*	
Breastfeeding plans	*n*	*2*	*3*	*46*	*60*	*36*	*38*
	Yes(%)	100%	66.7%	95.7%	98.3%	100%	100%
	No(%)	0	33.3%	4.3%	1.7%	0	0
	p-value	*0.36*		*0.41*		*ns*	
Planned breastfeeding period	*n*	*2*	*2*	*44*	*55*	*32*	*37*
	<6 months(%)	50%	0	11.4%	21.8%	18.8%	35.1%
	6-12 months(%)	0	50%	38.6%	36.4%	12.5%	10.8%
	>12-18 months(%)	0	0	4.5%	23.6%	6.2%	32.4%
	>18 months(%)	0	0	9.1%	7.3%	15.6%	5.4%
	Don't know(%)	50%	50%	36.4%	10.9%	46.9%	16.2%
	p-value	*0.37*		***0. 005***		***0.006***	

Supplementary table 5.1: Comparison of lifestyle behavior parameters of age group by stratifying trimester. Bold values: significant difference. ns: non-significant

Supplementary table 5.1:
Result: 2.6%, 57.7% and 39.7% of women were in the 1st, 2nd, 3rd trimester, respectively. Because of very few pregnant women in the 1st trimester group the statistical data might be chance findings. Significant difference of planned breastfeeding period was observed for the different age groups in 2nd and 3rd trimester. More women < 27 years of age were in doubt about planned breastfeeding period both in the 2nd trimester and 3rd trimester. No significant age difference for the alcohol intake, smoking status and plasma cotinine levels was observed for any trimesters.
Conclusion: After stratifying by the trimester, the age difference for women in the 2nd and 3rd trimesters was similar to the whole population. This is expected since most of women were in the 2nd and 3rd trimester at inclusion.

Parameter		1st trimester					2nd trimester					3rd trimester				
		North	Disko Bay	West	South	East	North	Disko Bay	West	South	East	North	Disko Bay	West	South	East
P-cotinine (ng/ml)	*n*	*1*	*0*	*4*	*0*	*0*	*7*	*30*	*65*	*6*	*1*	*5*	*18*	*46*	*4*	*2*
	Median	46.7		0.5			15.2	0.5	0.5	0.5	0.5	0.5	32.6	0.5	26.6	4.0
	Min-Max			0.5-52.8			0.5-129	0.5-184	0.5-190	0.5-116		0.5-160	0.5-144	0.5-190	0.5-126	0.5-7.5
	p-value			*0.29*					*0.46*					*0.43*		
Smoking status	*n*	*1*	*0*	*4*	*0*	*0*	*7*	*30*	*65*	*6*	*1*	*4*	*18*	*46*	*4*	*2*
	Current(%)	100%	0	50%	0	0	71.4%	46.7%	38.5%	16.7%	0	50%	72.2%	47.8%	50%	0
	Non-current(%)	0	0	50%	0	0	28.6%	53.3%	61.5%	83.3%	0	50%	27.8%	52.2%	50%	100%
	p-value			*0.36*					*0.25*					*0.25*		
Ever used cannabis	*n*	*1*	*0*	*4*	*0*	*0*	*5*	*29*	*63*	*6*	*1*	*5*	*16*	*39*	*4*	*2*
	Yes(%)	0	0	75%	0	0	60%	41.4%	39.7%	16.7%	0	40%	56.2%	56.4%	50%	0
	No(%)	100%	0	25%	0	0	40%	58.6%	60.3%	83.3%	100%	60%	43.8%	43.6%	50%	100%
	p-value			*0.58*					*0.58*					*0.58*		
Alcohol *During pregnancy*	*n*	*0*	*0*	*4*	*1*	*0*	*7*	*30*	*65*	*6*	*1*	*5*	*16*	*45*	*3*	*2*
	<1 time/month(%)	0	0	0	0	0	71.4%	100%	96.9%	83.3%	100%	100%	100%	97.8%	100%	100%
	1 time/month(%)	0	0	100%	100%	0	14.3%	0	3.1%	16.7%	0	0	0	2.2%	0	0
	2-3 time/month(%)	0	0	0	0	0	14.3%	0	0	0	0	0	0	0	0	0
	>1 time/week(%)	0	0	0	0	0	0	0	0	0	0	0	0	0	0	0
	p-value			*ns*					***0.006***					*0.97*		
Breastfeeding plans	*n*	*1*	*0*	*4*	*0*	*0*	*6*	*30*	*63*	*6*	*1*	*5*	*17*	*46*	*4*	*2*
	Yes(%)	100%	0	75%	0	0	66.7%	96.7%	100%	100%	100%	100%	100%	100%	100%	100%
	No(%)	0	0	25%	0	0	33.3%	3.3%	0	0	0	0	0	0	0	0
	p-value			*0.58*					***<0.0001***					*ns*		
Planned breastfeeding period	*n*	*1*	*0*	*3*	*0*	*0*	*4*	*29*	*59*	*6*	*0*	*5*	*17*	*41*	*4*	*2*
	<6 months(%)	0	0	33.3%	0	0	0	31%	11.9%	16.7%	0	40%	23.5%	26.8%	50%	0
	6-12 months(%)	100%	0	0	0	0	50%	34.5%	37.3%	50%	0	0	0	17.1%	25%	0
	>12-18months(%)	0	0	0	0	0	0	17.2%	15.3%	16.7%	0	40%	17.6%	19.5%	0	50%
	>18 months(%)	0	0	0	0	0	0	0	11.9%	0	0	0	5.9%	12.2%	25%	0
	Don't know(%)	0	0	66.7%	0	0	50%	17.2%	23.7%	16.7%	0	20%	52.9%	24.4%	0	50%
	p-value			*0.14*					*0.10*					*0.46*		

Supplementary table 5.2: Comparison of lifestyle behavior parameters of regions by stratifying trimester. Bold values: significant difference. ns: non-significant

Supplementary table 5.2:

Result: 2.6%, 57.7% and 39.7% of women were in the 1st, 2nd, 3rd trimester, respectively. Because of very few pregnant women in the 1st trimester group the statistical data might be chance findings. After stratifying by the trimester, significant regional differences of alcohol intake during pregnancy and breastfeeding plans were observed for pregnant women in the 2nd trimester. The women in the 2nd trimester from, *North*, *South* and *West* had higher alcohol intake during pregnancy. Pregnant women from *North* had higher percent to say "no" to breastfeeding plans.

Conclusion: The regional difference were similar to the whole population for women in the 2nd trimesters, suggesting the data of 2nd trimester influenced the whole dataset, because 58% women were in the 2nd trimester at inclusion.

Abbreviations and glossary

ACCEPT	**A**daption to **C**limate **C**hange, **E**nvironmental **P**ollution, and Dietary **T**ransition
Blubber	The fat of marine mammals, which are eaten as delicacy in Greenland
BMI	Body Mass Index
CVD	Cardiovascular diseases
DM2	Diabetes mellitus type 2
Fatty acids	Essential nutrient group, which is saturated or unsaturated, the latter can be mono- or polyunsaturated. Two polyunsaturated fatty acids to note are:<br>*n – 3 fatty acid*: mainly dominant in animals, e.g. marine food (high levels in Arctic populations)<br>*n – 6 fatty acid*: generally found in the herbal environment (high levels in Western populations)
FBDGs	Food-based dietary guidelines
Fetal programming	Concept that links environmental conditions during embryonic and fetal development with risk of diseases later in life
FFQ	Food frequency questionnaire
Inuit	Indigenous people inhabiting in the Arctic area
Mattak	The skin of the marine mammals, which are eaten as delicacy in Greenland
OCP	Organochlorine pesticide (legacy POP **-** use and emission was banned in the 1970's)
PCB	Polychlorinated biphenyls (legacy POP **-** use and emission was banned in the 1970's)
PFAS	Perfluoroalkylated substances<br>(Perfluorinated compound. Emerging and current used POP)
PFCA	Perfluorocaboxylated acid<br>(Perfluorinated compound. Emerging and current used POP)
PFSA	Perfluorosulfonated acid<br>(Perfluorinated compound. Emerging and current used POP)
POPs	Persistent organic pollutants
MetS	Metabolic syndrome
WHO	World Health Organization
